# Bio-Mechanical Model of Osteosarcoma Tumor Microenvironment: A Porous Media Approach

**DOI:** 10.3390/cancers14246143

**Published:** 2022-12-13

**Authors:** Yu Hu, Navid Mohammad Mirzaei, Leili Shahriyari

**Affiliations:** Department of Mathematics and Statistics, University of Massachusetts Amherst, Amherst, MA 01003, USA

**Keywords:** osteosarcoma, cancer modeling, tumor microenvironment, partial differential equation, tumor growth, poroelasticity, Biot equations

## Abstract

**Simple Summary:**

Osteosarcoma is the most common type of bone cancer seen in children to young adults with poor prognosis. To find effective treatments, it is crucial to understand the mechanism of the initiation and progression of the osteosarcoma tumors. In this paper, we introduce a PDE model for the progression of osteosarcoma tumors by considering the location of different cell types, including immune and cancer cells, in the tumors. We perform several simulations using the developed model to investigate the importance and role of the different immune cells’ location in the growth of the tumors. The results show that the co-localization of macrophages and cancer cells promotes tumors’ growth.

**Abstract:**

Osteosarcoma is the most common malignant bone tumor in children and adolescents with a poor prognosis. To describe the progression of osteosarcoma, we expanded a system of data-driven ODE from a previous study into a system of Reaction-Diffusion-Advection (RDA) equations and coupled it with Biot equations of poroelasticity to form a bio-mechanical model. The RDA system includes the spatio-temporal information of the key components of the tumor microenvironment. The Biot equations are comprised of an equation for the solid phase, which governs the movement of the solid tumor, and an equation for the fluid phase, which relates to the motion of cells. The model predicts the total number of cells and cytokines of the tumor microenvironment and simulates the tumor’s size growth. We simulated different scenarios using this model to investigate the impact of several biomedical settings on tumors’ growth. The results indicate the importance of macrophages in tumors’ growth. Particularly, we have observed a high co-localization of macrophages and cancer cells, and the concentration of tumor cells increases as the number of macrophages increases.

## 1. Introduction

Osteosarcoma (also called osteogenic sarcoma) is the most common type of cancer that starts in the bones. It happens most often in children, adolescents, and young adults. Approximately 800 new cases of osteosarcoma are reported each year in the U.S.; of these cases, about 400 are in children and teens [1]. Surgery, chemotherapy, radiation therapy, and targeted therapy are the types of standard treatment for osteosarcoma [2].

There are many mathematical models to understand and investigate biomedical problems such as cancer. Mainly, bio-mechanical models are developed to investigate the spatial interaction among cells and their movements in tumors using appropriate physical laws [3,4]. In one study, the authors have developed a partial differential equation (PDE) model to study the breast tumors’ progression in mice as a fluid structure because the breast tumors are mainly confined in the mammary gland [5]. However, when the tissues are porous, modeling with techniques of porous media can be more realistic [6,7,8,9,10,11]. Although the Biot equations are the critical part of poroelasticity theory, few studies have incorporated the equations for porous media (the Biot equations) into their model.

**Figure 1:**
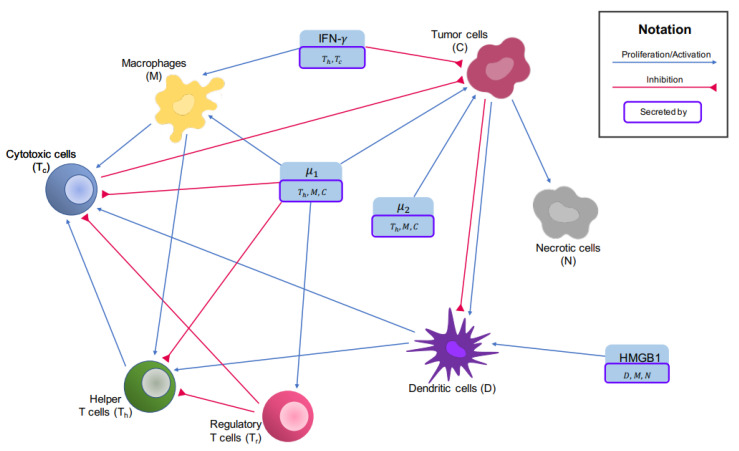
Interaction network of the tumor microenvironment in osteosarcoma. Blue arrows show activation and proliferation while inhibitions are indicated by red arrows.

There is a limited number of mathematical models for osteosarcoma, and to the best of our knowledge, there is no PDE model for osteosarcoma. To find optimal treatments for osteosarcoma patients based on their immune profiles, an ODE model has been recently developed to study the key players in the tumors’ microenvironment and their interaction network, which are shown in Figure 1. In this model, osteosarcoma tumors have been grouped based on their immune profile. For each group, the models’ parameters that could not be found in current literature have been estimated using the tumors’ gene expression profiles [12,13].

Here, we propose a bio-mechanical multiphase model that describes the dynamics of the tumor microenvironment of osteosarcoma. The model PDE consists of two parts: the biological part and the mechanical part. The biological part is an extension of the ODE system provided in [12] to a system of RDA equations. The coupling terms from the mechanical part are the fluid velocity involved in the convection terms. The mechanical part is derived from the first principles, following the same argument that leads to Biot equations. Using the mechanical part, we model the motion of the fluid, which carries various kinds of cells and chemokines, and is an essential part of the continuity equation as fluid flow through porous media. The coupling term from the biological part is the additional strain of the solid tumor caused by the increase or reduction of cells. We investigate the reference case in the absence of influx and cases with different settings to examine the importance of considering cell locations and their movements in the mathematical modeling of the tumors’ growth.

## 2. Materials and Methods

### 2.1. Tumor Microenvironment and Interaction Network

The key players of the tumor microenvironment in osteosarcoma and their interaction network are shown in Figure 1, which lay the foundation of the ODE paper [12].

The vector, $\left[X\right]=(\left[X_{1}\right],\left[X_{2}\right],\ldots ,\left[X_{14}\right])$ represents the 14 key players we will investigate. The values of $\left[X_{i}\right]$’s are re-scaled by the corresponding steady-state abundance of cells or cytokines. The correspondence of $\left[X_{i}\right]$’s to the cells or molecules and the steady-state abundance values are shown in Table 1.

### 2.2. Biological Part of the Model

We adopt the Reaction-Diffusion-Advection equations by adding the diffusion term $D_{cell}\Delta \left[X_{i}\right]$ and the advection term $b\nabla \cdot (\left[X_{i}\right]\kappa \nabla p)$ to the ODEs of each cell type. Here, $D_{cell}$ is the diffusion coefficient of cells, i=2,4,5,6,7,8,9, *b* is the advection coefficient which is set to be 1 for the sake of simplicity, and κ is the hydraulic conductivity of the solid tumor.The derivation of the form of the advection term will be explained in detail in the mechanical modeling part. We assume that necrotic cells are immotile, so we do not consider any diffusion or advection for them [14]. As for the naive T-cells and naive macrophages, since they activate mainly outside of the tumor microenvironment [15,16,17], we are only interested in their level, not their spatial distributions. We, therefore model their dynamics via ODEs.

We add the diffusion and advection terms to every ODE of cytokines. We denote the diffusion coefficient of HMGB1 (*H*) by $D_{H}$ and the diffusion coefficients of other cytokines ($I_{\gamma },\mu_{1},\;\mathrm{and}\;\mu_{2}$ ) by $D_{cyto}$.

In summary, the biological part of our model can be written as

(1) $$\frac{\partial [X_{i}]}{\partial t}-D_{i}\Delta \left[X_{i}\right]+b_{i}\nabla \cdot \left(\left[X_{i}\right]\kappa \nabla p\right)=f_{i},\;i=1,\ldots ,14,$$

where $D_{i}$ is $0,D_{cell},D_{cyto},\;\mathrm{or}\;D_{H}$, depending on the specific equation, $b_{i}=0\;\mathrm{or}\;1$, and $f_{i}$’s model the biochemical interactions which are given in [12] (please see Appendix A). Consequently, the equations can be written in the following vector form

(2) $$\frac{\partial [X]}{\partial t}-D\Delta \left[X\right]+B\nabla \cdot \left(\left[X\right]\kappa \nabla p\right)=f,$$

where $D=\mathrm{diag}\{D_{i}\}$ and $B=\mathrm{diag}\{b_{i}\}$ are diagonal coefficient matrices.

### 2.3. Mechanical Part of the Model

In the chapter “Cancer Models and Their Mathematical Analysis” of the book [14], Friedman utilizes the continuity equation that generally takes the form

(3) $$\frac{\partial \rho }{\partial t}+\nabla \cdot \left(\rho v\right)=f,$$

where ρ is the density of the matter of interest, v is usually the fluid velocity, and *f* is the external source. In the context of cancer modeling, v represents the continuous motion of cells within the tumor. Later, Friedman specifies the meaning of v to be the velocity of the fluids that flow through porous media and applies Darcy’s law, treating the tumor tissue as a porous medium and assuming the moving cells are carried with the flow.

Here, we will go one step further and take advantage of the full poroelasticity equations that cover Darcy’s law while maintaining a variable that describes the movement of the solid body—the tumor. Therefore, we put the description of fluid flow into the theory of porous media and obtain a source for the solid/mesh movement.

#### 2.3.1. Governing Equations

We start with presenting the stress($\sigma_{ij}$)-strain($e_{ij}$) relation for linear, isotropic poroelastic material. In the spirit of Hooke’s law, the linear relation is

(4) $$\begin{array}{c} \sigma_{ij}=\left(K_{u}-\frac{2G}{3}\right)\delta_{ij}e+2Ge_{ij}-\alpha \delta_{ij}\zeta . \end{array}$$

Here, $e_{ij}=\frac{1}{2}\left(\frac{\partial u_{i}}{\partial x_{j}}+\frac{\partial u_{j}}{\partial x_{i}}\right)$ is the stress, $e=e_{ii}=\frac{\partial u_{1}}{\partial x_{1}}+\frac{\partial u_{2}}{\partial x_{2}}=\nabla \cdot u$ is the total volumetric strain, where u is the solid displacement of the material, $\delta_{ij}$ is the Kronecker delta, ζ is the fluid content, $K_{u}$ is the undrained bulk modulus, *G* is the shear modulus, and α is the Biot effective stress coefficient. The total stress comprises two parts, the contribution from the displacement of the solid framework and the contribution from the fluid flow. In our case, when building a multiphase model, we consider the contribution from the variation of the number of cells that constitute the solid frame of the tumor, namely, the cancer cells *C*, the necrotic cells *N*, the naive dendritic cells $D_{n}$, and the dendritic cells *D*, since most tumors are infiltrated by dendritic cells [18].

The expression for the stress can be formally written as

stress=solidcontribution+fluidcontribution+biologicalcontribution.

For the biological contribution, we apply Hooke’s law again. The spring constant is the drained bulk modulus *K*, and the distance is induced by the volumetric change resulting from the variations of cells’ number. The number of cells changes with respect to time in a pattern that we have already given:

(5) $$\frac{\partial [X_{i}]}{\partial t}-D_{i}\Delta \left[X_{i}\right]+b_{i}\nabla \cdot \left(\left[X_{i}\right]v_{f}\right)=f_{i}.\;i=1,\ldots ,14.$$

The volume of cells that are a part of the solid tumor *V* is calculated through

(6) $$V=\sum_{i=7}^{10}A_{X_{i}}\left[X_{i}\right]$$

where $A_{X_{i}}$ is the size of the cell $X_{i}$. The variable $X_{i}$ when i=7,8,9,and10 corresponds to naive dendritic cells, dendritic cells, cancer cells, and necrotic cells. To summarize, if we regard the tumor as isotropic, the stress-strain relation for the tumor is,

(7) $$\begin{array}{c} \sigma_{ij}=\left(K_{u}-\frac{2G}{3}\right)\delta_{ij}e+2Ge_{ij}-\alpha \delta_{ij}\zeta -KV\delta_{ij}. \end{array}$$

This expression is verified in the paper by Roose et al. [19], where they have used the same form. Here, we have the density of cells at any time from solving the biological equations, so we can calculate the volumetric change without resorting to the other given formulae of tumor growth. We complete the set of governing equations with the following ones.

Definition of strain:

(8) $$e_{ij}=\frac{1}{2}\left(\frac{\partial u_{i}}{\partial x_{j}}+\frac{\partial u_{j}}{\partial x_{i}}\right),$$

where we define u as the solid displacement of the solid tumor, a macroscopic measure.

Constitutive equation of the fluid pressure *p*:

(9) $$\begin{array}{cc} p & =M(\zeta -\alpha e), \end{array}$$

where *M* is the Biot modulus.

Equilibrium equation (Newton’s law of motion):

(10) $$\frac{\partial \sigma_{ij}}{\partial x_{j}}=0.$$

Darcy’s law (flow of a fluid with constant viscosity across a solid is a function of its pressure difference and the solid properties):

(11) $$q_{i}=-\kappa \frac{\partial p}{\partial x_{i}}.$$

Here, q is the fluid-specific discharge vector, defined as the volume of fluid passing through a unit area of the porous medium, per unit time, in the direction normal to the area, which has the dimension of velocity, [L/T], and κ is the hydraulic conductivity.

Continuity equation (conservation of mass):

(12) $$\frac{\partial \zeta }{\partial t}+\frac{\partial q_{i}}{\partial x_{i}}=0.$$

Solving the governing Equations (7)–(12) simultaneously, we obtain the mechanical part of our model: In Ω(t)

(13) $$\begin{array}{cc} -\left(K-\frac{2G}{3}\right)\nabla \left(\nabla \cdot u\right)-G\Delta u+\alpha \nabla p+K\nabla V & =0, \end{array}$$

(14) $$\begin{array}{cc} \frac{\partial }{\partial t}\left(c_{0}p+\alpha \nabla \cdot u\right)-\kappa \Delta p & =0, \end{array}$$

which is to be aligned with the biological part (1)

(15) $$\frac{\partial [X_{i}]}{\partial t}-D_{i}\Delta \left[X_{i}\right]+b_{i}\nabla \cdot \left(\left[X_{i}\right]\kappa \nabla p\right)=f_{i},\;i=1,\ldots ,14.$$

The parameter values of the local dynamics f of the biological part are shown in [12] and other parameter values are shown in Table 2.

#### 2.3.2. Initial and Boundary Conditions

For the mechanical part, for simplicity, we set the initial displacement field to zero and the interstitial fluid pressure equal to the blood pressure everywhere. This yields

(16) $$\begin{array}{cc} u(x,0) & =(0,0),\\ p(x,0) & =p_{0}, \end{array}$$

where $p_{0}$ is the average blood pressure of humans. Since we set u at time 0 as the reference, we shall reset the biological coupling term in Equation (13) as $K\nabla V^{*}=K\nabla \left(V-V_{0}\right)$ where $V_{0}$ is V(t=0) so that at every time, *V* is to be compared with $V_{0}$. At t=0, it can be seen that u=0; hence compatibility is ensured. The initial conditions of the biological part can be found in [12]; we have also listed them here in Table A1.

We use the no-flux boundary condition for u, in which case the tumor can grow freely while ignoring mechanical influences from surrounding tissues. We also treat the boundary of the tumor as the boundary of the normal tissues and set the pressure to be fixed as the blood pressure.

(17) $$\begin{array}{cc} \frac{\partial u(x,t)}{\partial n} & =0,\;\mathrm{on}\;\partial \Omega , \end{array}$$

(18) $$\begin{array}{cc} p(x,t) & =p_{0},\;\mathrm{on}\;\partial \Omega , \end{array}$$

where n is the outward unit normal vector. To exclude translation or rotation of the domain, two constraints are added [24]

(19) $$\int_{\partial \Omega }udx=0,\;\int_{\partial \Omega }u\times ddx=0,$$

where d is the deformation vector.

We will apply both the no-flux boundary conditions and the Robin Boundary conditions to the biological equations. A no-flux boundary condition resembles the case when the tumors develop without interference, while the Robin boundary condition imitates the case when immune cells infiltrate. They can be written as

(20) $$\begin{array}{cc} \frac{\partial [X_{i}]}{\partial n}=0, & \;\mathrm{on}\;\partial \Omega ,\\ \mathrm{or}, & \end{array}$$

(21) $$\begin{array}{cc} \frac{\partial [X_{i}]}{\partial n}+\alpha_{i}\left(\left[X_{i}\right]-\left[\hat{X_{i}}\right]\right)=0, & \;\mathrm{on}\;\partial \Omega , \end{array}$$

where $\alpha_{i}$ is the influx rate and is only non-zero for immune cells. The quantity $\left[{X_{i}}^{*}\right]$ pertains to the maximum levels of immune cells in lymph nodes and blood.

### 2.4. Weak Formulation and Discretization

#### 2.4.1. Weak Formulation

We first rewrite the biological equations in the Eulerian system. Noticing that the definition of the material derivative is given by

(22) $$\frac{D}{Dt}=\frac{\partial }{\partial t}+v_{f}\cdot \nabla ,$$

we have

(23) $$\begin{array}{cc} \; & \frac{\partial [X_{i}]}{\partial t}-D_{i}\Delta \left[X_{i}\right]+b_{i}\nabla \cdot \left(\left[X_{i}\right]v_{f}\right)\\ = & \frac{\partial [X_{i}]}{\partial t}-D_{i}\Delta \left[X_{i}\right]+b_{i}\nabla \left[X_{i}\right]\cdot v_{f}+b_{i}\left[X_{i}\right]\nabla \cdot v\\ = & \frac{D[X_{i}]}{Dt}-D_{i}\Delta \left[X_{i}\right]+b_{i}\left[X_{i}\right]\nabla \cdot v_{f}=f_{i}, \end{array}$$

where $v_{f}$ is the fluid velocity. Let I=(0,T], $V={\left(L^{2}\left(I;H^{1}\left(\Omega \right)\right)\right)}^{3}$, $Q=L^{2}\left(I;H^{1}\left(\Omega \right)\right)$, $X={\left(L^{2}\left(I;H^{1}\left(\Omega \right)\right)\right)}^{14}$, and $a\left(u,v\right)=\left(\left(K-\frac{2G}{3}\right)\nabla \cdot u,\nabla \cdot v\right)+\left(G\nabla u,\nabla v\right)$, the weak formulation of the problem is: find (u,p,[X])∈V×Q×X that satisfies (16) and Lagrange multipliers $\lambda_{i}$ such that for a.e. t∈(0,T]

(24) $$\begin{array}{cc} a\left(u,v\right)+\left(\alpha \nabla p,v\right)+\left(K\nabla V^{*},v\right)-\sum_{i=1}^{3}\lambda_{i}\left(v,z_{i}\right)-\sum_{i=1}^{3}\omega_{i}\left(u,z_{i}\right) & =0,\; \end{array}$$

(25) $$\begin{array}{cc} \left(c_{0}\frac{\partial p}{\partial t},q\right)+\left(\alpha \frac{\partial (\nabla \cdot \;u)}{\partial t},q\right)+\left(\kappa \nabla p,\nabla q\right) & =0,\; \end{array}$$

and

(26) $$\begin{array}{cc} \left(\frac{D[X]}{Dt},\Xi \right)+\left(D\nabla [X],\nabla \Xi \right)+\left(b[X]\kappa \Delta p,\Xi \right) & =(f,\Xi ),\; \end{array}$$

for all appropriate choices of test functions v,q and Ξ. The last two terms at the left-hand side of Equation (24) are added to exclude rigid body movements, with the following constraints of Lagrange multipliers $\lambda_{i}$ and test functions $\omega_{i}$ corresponding to the bases $z_{i}$ of the space of rigid body movements:

(27) $$\sum_{i=1}^{3}\lambda_{i}\left(v_{h},z_{i}\right)+\sum_{i=1}^{3}\omega_{i}\left(u_{h},z_{i}\right)=0,$$

where $z_{i}\in \left\{\left(1,0\right),\left(0,1\right),\left(-x_{2},x_{1}\right)\right\}$.

#### 2.4.2. Discretization

We only list the result of discretization in this section. The details about discretization are given in Appendix B.

Denote by $V_{h}$, $Q_{h}$, $X_{h}$ the appropriate finite dimensional subspaces of V, Q, X, respectively, and denote the time step size by *k*. Let $u^{n}=u\left(t_{n}\right)$ and $p^{n}=p\left(t_{n}\right)$ and let $U^{n}$ and $P^{n}$ be their approximations, respectively. We use Lagrange elements for the two mechanical equations. The fully discrete approximation for the mechanical problem is: find $U^{n}\in V_{h}$ and $P^{n}\in Q_{h}$ for n=1,2,…,N, and Lagrange multipliers $\lambda_{i}$ such that

(28) $$\begin{array}{cc} a\left(U^{n},v_{h}\right)+\left(\nabla P^{n},v_{h}\right)+\left(K\nabla V^{*n},v_{h}\right)-\sum_{i=1}^{3}\lambda_{i}\left(v_{h},z_{i}\right)-\sum_{i=1}^{3}\omega_{i}\left(u_{h},z_{i}\right)=0,\; & \end{array}$$

(29) $$\begin{array}{cc} k^{-1}\left(c_{0}\left(P^{n}-P^{n-1}\right),q_{h}\right)+k^{-1}\left(\alpha \nabla \cdot \left(U^{n}-U^{n-1}\right),q_{h}\right)+\left(\kappa \nabla P^{n},\nabla q_{h}\right)=0,\; & \end{array}$$

for all basis functions $v_{h}$ and $q_{h}$ of the spaces $V_{h}$ and $Q_{h}$.

After solving the mechanical problem (28) and (29), we obtain the mesh displacement vector $U^{n}$. We can apply it to the domain if we want to observe the shape and size of the domain with respect to the reference frame, at any time.

We then solve the biological problem. We use a mixed finite element space enriched with bubble elements for the biological equations. The fully discrete approximation for the biological problem is: find $X^{n}\in Q_{h}$ for n=1,2,…,N, such that

(30) $$k^{-1}\left({\left[X\right]}^{n}-{\left[X\right]}^{n-1},\Xi_{h}\right)+\left(D\nabla {\left[X\right]}^{n},\nabla \Xi_{h}\right)+\left(b{\left[X\right]}^{n}\kappa \Delta p,\Xi_{h}\right)=(f,\Xi_{h})$$

for all basis functions $\Xi_{h}$ of the space $X_{h}$.

## 3. Results

There are three distinct groups of osteosarcoma tumors based on their immune profile [25]. The recent ODE model [12] investigates the dynamics of key players given in Table 1 for each of these three groups by finding their parameter values using tumors’ gene expression profiles. In this paper, we have used the parameter values for the biochemical interactions ($f_{i}$’s) obtained for the first group of osteosarcoma tumors using the TARGET data with 88 samples (downloaded from the UCSC Xena web portal [26]). We simulated several scenarios to evaluate the model’s performance and gather insights about the role of the spatial interactions among key players and their movements during the tumors’ growth. We performed four main categories of simulations: the reference case Section 3.1, with a different initial profile Section 3.2, with immune cells infiltration from the boundary Section 3.3, and with external sources Section 3.4, Section 3.5, Section 3.6 and Section 3.7, as shown below.

### 3.1. The Reference Case

The reference case has the simplest settings. In this case, the initial conditions of the biological Equations (2) are uniform throughout the domain with no-flux boundary condition, meaning that there is no infiltration of cells. Finally, there is no alteration of biological coefficients, i.e., no treatments are modeled. Mathematically, we solve the Equations (13)–(15) with the boundary conditions:

(31) $$\begin{array}{cc} t(x,t)\cdot n & =0,\;\mathrm{on}\;\partial \Omega , \end{array}$$

(32) $$\begin{array}{cc} p(x,t) & =p_{0},\;\mathrm{on}\;\partial \Omega , \end{array}$$

(33) $$\begin{array}{cc} \frac{\partial [X]}{\partial n} & =0,\;\mathrm{on}\;\partial \Omega , \end{array}$$

and initial conditions:

(34) $$\begin{array}{cc} u(x,0) & =(0,0), \end{array}$$

(35) $$\begin{array}{cc} p(x,0) & =p_{0}, \end{array}$$

(36) $$\begin{array}{cc} \left[X](x,0\right) & =C_{i}, \end{array}$$

where $C_{i}$, for i=1,2,or3 is the vector of initial condition for 3 clusters, shown in Table A1. The domain Ω is initially a circle with a diameter 0.01 (m).

**Figure 2:**
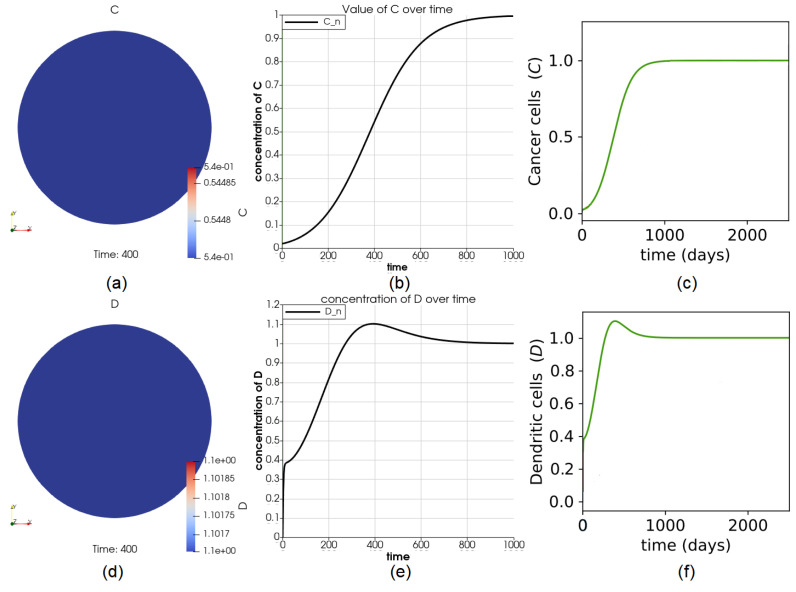
Dynamics of variables *C* and *D*. Part (**a**) shows the value of *C* at time t=400 for the first group of osteosarcoma patients provided in [12]. The agreement of the curve in (**b**) with the green curve (results of the first group of tumors) from (**c**) shows that the dynamics of *C* at any point in the domain match the solution from the ODE paper. Sub-figure (**d**–**f**) show the dynamics of *D*.

As expected, the number of cells is the same everywhere throughout the domain at any time for all variables because the initial values of variables are uniform throughout the entire domain. The values of *C* and *D* in the entire domain are shown in Figure 2a,d. The perfect agreement between the results of the reference case (Figure 2a,e and the ones obtained from the ODE model (green curves in Figure 2c,f) provide validation for the PDE model and its implementation.

**Figure 3:**
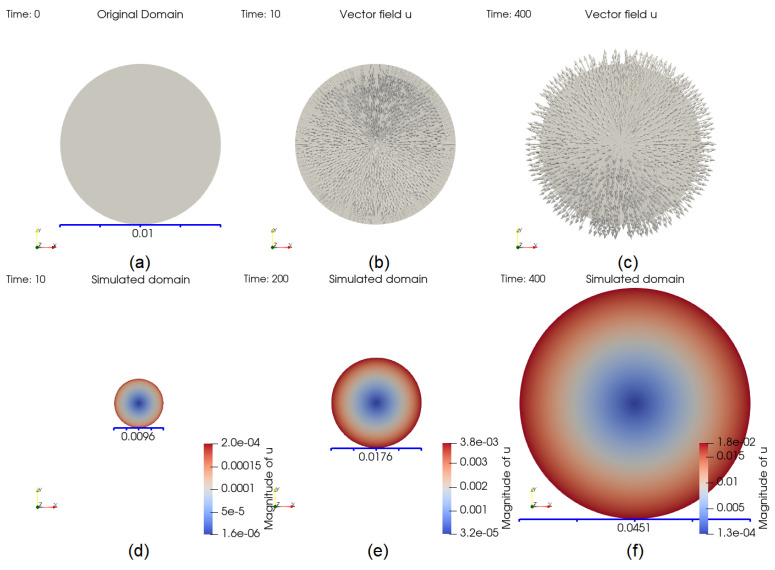
Evolution of the domain. The original domain is plotted in (**a**). At t=10, the arrows, showing the vector field u, are pointing inward, since *V* is decreasing in (**b**) whereas in (**c**), they are pointing outward since *V* is increasing, which means that the domain is growing at t=400. Part (**d**–**f**) show the size of the domain at time t=10, 200, and 400, respectively.

The size change of the domain is partly displayed in Figure 3. At t=10, the number of cells *V* decreases, implying that the size should decrease accordingly. The inward pointing displacement vector field u shown in Figure 3b suggests the same trend. An opposite example is given in Figure 3c, for t=400. The domain at t=10, 200 and 400 are shown in the same frame in Figure 3d–f, respectively.

The solution of the reference case is essential because it can be used to validate the model and compare the results of different scenarios with the reference case. We calculate the total number of cells that are a part of the solid tumor: cancer cells, necrotic cells, dendritic and naive dendritic cells. We aim to simulate the tumor’s size changes, as indicated by the number of cells, by applying the solid displacement vector u to the domain. Because the domain is selected to be the tumor or part of it, the solid displacement vector u tells us how each point on the solid tumor moves with respect to the reference frame at any time.

We use the initial total number of cells $V_{0}$ and the initial diameter $d_{0}=0.01\left(m\right)$ as references. If, at one time, the total number of cells *V* reaches $nV_{0}$, we have an intuitive but crude estimate of the tumor size at that time, $A=nA_{0}=\frac{\pi }{4}\times 10^{-4}n\left(m^{2}\right)$, and an estimate of the diameter of the tumor $d=\sqrt{n}d_{0}$ (since we are working on 2D cases). For example, by the time the total number of cells becomes $4V_{0}$, we expect the diameter to reach approximately 0.02 (m). In fact, the size can be bigger because of the remnants of dead cells inside the tumor or stronger angiogenesis, or it can be smaller if the squeezing effect dominates. In [27], the authors have built a generalized linear model that connects the tumor cell number with cell diameter and tumor diameter after studying 38 tumor samples with $R^{2}=0.92$. The relation is

(37) $$\mathrm{No}.\;\mathrm{of}\;\mathrm{cells}/\mathrm{colony}=2.40\frac{{\left(\mathrm{colony}\;\mathrm{diameter}\right)}^{2.378}}{{\left(\mathrm{cell}\;\mathrm{diameter}\right)}^{2.804}}.$$

**Figure 4:**
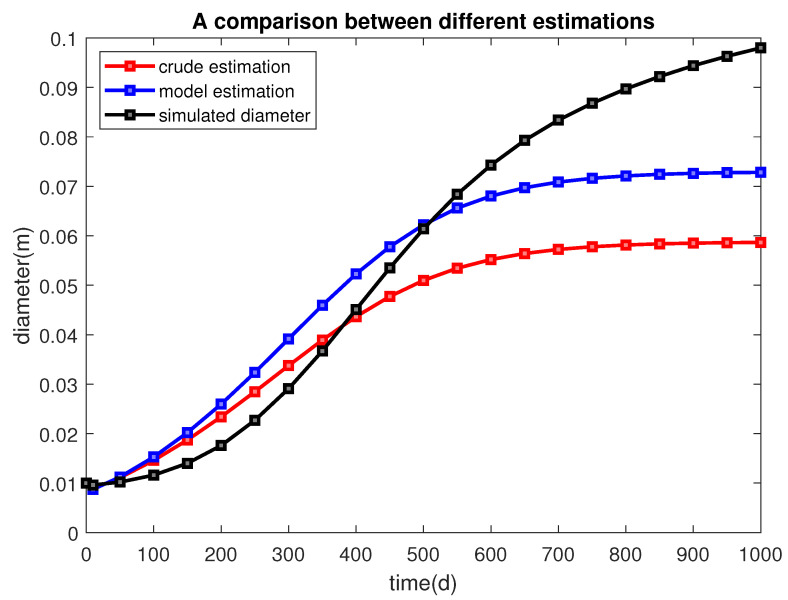
The diameter of the tumor is estimated from different sources. The red line, blue line, and black line represent the estimation from taking a square root, a linear model, and applying the displacement vector. It can be seen that the estimate obtained from applying the displacement vector is sound.

Using this formula, we obtain another estimate of the tumor’s diameter (Table 3). Figure 4 provides a visualized comparison between different estimates. The number of cells decreases in the first 17 days due to a sharp reduction of necrotic cells, then increase rapidly, and finally remains unchanged at about 1100 days. We, therefore, can estimate the evolution of a biological feature, the size of the tumor, using a calculated mechanical quantity, the displacement vector.

### 3.2. The Case of a High Level of M and $T_{c}$ in the Middle of the Tumor at the Initial Time

**Figure 5:**
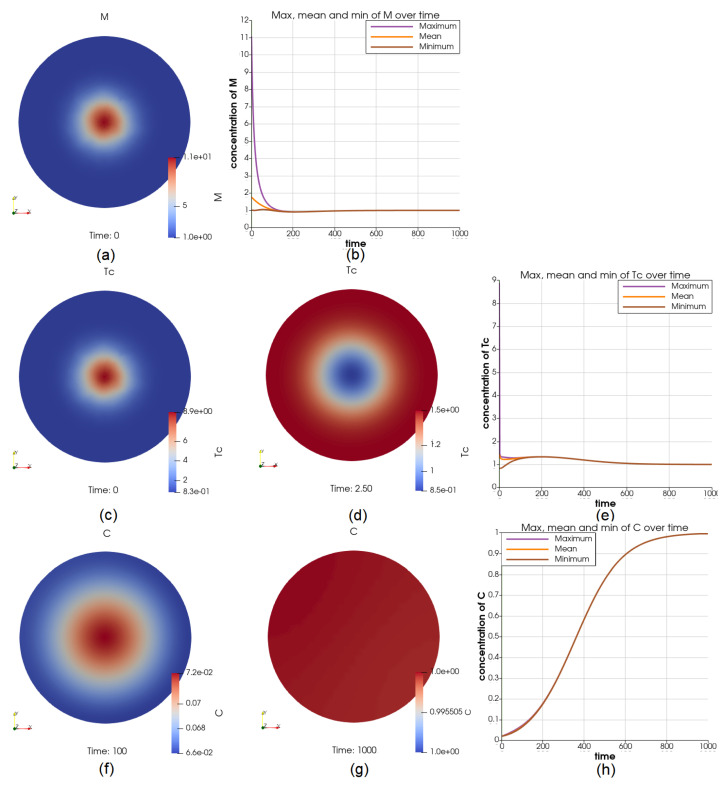
Illustrations for the case of more *M* and $T_{c}$ in the middle initially. Sub-figure (**a**,**c**) shows the initial value of $T_{c}$ and *M* through the domain, respectively; (**d**) shows $T_{c}$ at t=2.5, which marks a drastic change in both the profile and values; the dynamics of *M* and $T_{c}$ over the whole time interval is shown in (**b**,**e**), respectively; the cancer cell concentrations at t=100 and t=1000 are shown in (**f**,**g**), and the dynamics of *C* is shown in (**h**).

This is to imitate the condition of a high initial biological activity in the middle of the tumor. The initial concentration of *M* and $T_{c}$ in the center is set to be 11 times higher than the boundary, as shown in Figure 5a,c. The distribution of $T_{c}$ over the domain rapidly changes, as we can see from Figure 5d at t=2.5. The concentration of $T_{c}$ becomes higher on the boundary, and the overall concentration greatly decreases. However, *M* maintains the “higher concentration in the middle” profile throughout the process. The dynamics of *M* and $T_{c}$ over the domain are displayed in Figure 5b,e. We can see that the initial differences last for less than 200 days. At t=200, $V=6.25V_{0}$ (comparing with the reference case when $V=5.48V_{0}$), *C* has higher concentration in the middle and ranges between 0.177 and 0.179, Figure 5f. At t=1000, $V=34.98V_{0}$ (comparing with the reference case when $V=34.6V_{0}$), the distribution of *C* is close to uniform, Figure 5g. The initial differences in *M* and $T_{c}$ have not brought significant different behaviors in *C*. The steady-state value is still 1.

### 3.3. The Case of Using a Robin Boundary Condition for Five Types of Cells

**Figure 6:**
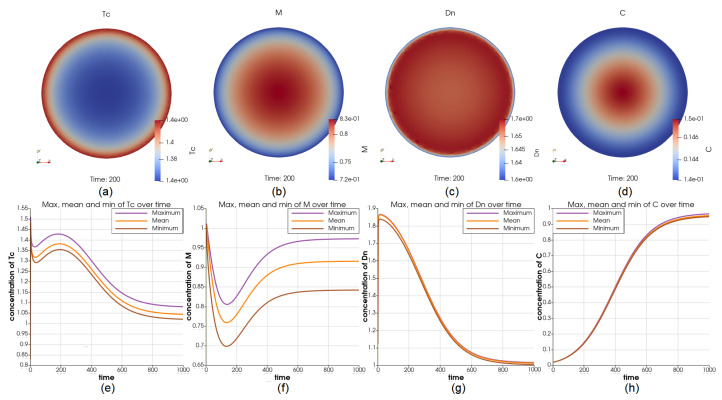
Illustrations for the case of using Robin boundary condition for 5 immune cell types. Sub-figure (**a**–**d**) show the value of $T_{c}$, *M*, $D_{n}$, and *C* at t=200; Sub-figure (**e**–**h**) show the maximum and minimum in the whole interval for $T_{c}$, *M*, $D_{n}$, and *C*, respectively. Using the same boundary condition for 5 immune cell types results in different profiles for these cell types.

To simulate the movements of the immune cell types: *M*, $T_{c}$, $T_{h}$, $T_{r}$, and $D_{n}$ between the inside and outside of the tumor, we use the uniform initial value on the entire domain for all cell types and Robin boundary condition by assigning $\alpha_{i}=100$ and $\alpha_{i}\left[{\hat{X}}_{i}\right]=1$ for these five immune cell types. Although we used the same boundary condition for all these cells, it has resulted in different distributions of $T_{c}$, *M*, and $D_{n}$ over the domain at t=200, respectively (Figure 6a–c). The value of *M* over the domain varies greatly while $D_{n}$ becomes nearly uniform after t=100. At t=200, $V=5.09V_{0}$ (comparing with the reference case when $V=5.48V_{0}$), *C* has lower concentration over the boundary and ranges between 0.143 and 0.147 (lower than 0.16 in the reference case), see Figure 6d. At t=1000, $V=33.04V_{0}$ (comparing with the reference case when $V=34.6V_{0}$), the dynamics of *C* at the center and on the boundary are displayed in Figure 6h. In this case, we observe that the cells that activate the growth of cancer cells are outperformed by cells that inhibit the growth of cancer cells. From applying the Robin boundary condition to every cell type one at a time, we observe that the changes in macrophages result in the biggest changes in the concentration of cancer cells compared to the other immune cell types.

### 3.4. The Case of a Positive Source of M in the Middle

**Figure 7:**
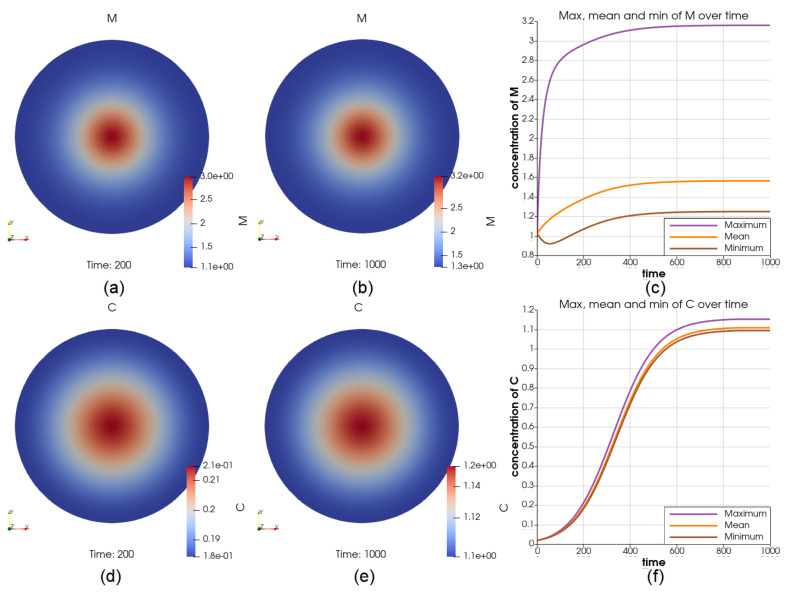
Illustrations for the case of a source of *M* in the middle. Sub-figure (**a**,**b**) show the value of *M* at t=200 and t=1000, respectively; (**c**) shows the dynamics of the maximum and minimum of *M*, from where we can see that the source has introduced a significant amount of increase in the middle; the cancer cell concentration at t=200 and t=1000 are shown in (**d**,**e**); the value of the maximum and minimum of *C* over the whole time interval is shown in (**f**). These figures suggest that the cancer cells will be at the place where there are more macrophages, yet the concentration still reaches a steady state.

We simulated the case of adding a constant source of *M* at all times in the middle of the tumor. The source has resulted in 200% higher concentration of *M* in the middle of the tumor for most of the time interval, see Figure 7a–c. As a result, at t=200, $V=6.70V_{0}$ (comparing with the reference case when $V=5.48V_{0}$), *C* has 20% higher concentration in the middle and ranges between 0.18 and 0.21, see Figure 7d. Moreover, at t=1000, $V=38.00V_{0}$ (comparing with the reference case when $V=34.6V_{0}$), *C* has a higher concentration in the middle and ranges between 1.08 and 1.15, see Figure 7e. The dynamics of *C* at the center and on the boundary are displayed in Figure 7f.

### 3.5. The Case of a Positive Source of $T_{c}$ in the Middle

**Figure 8:**
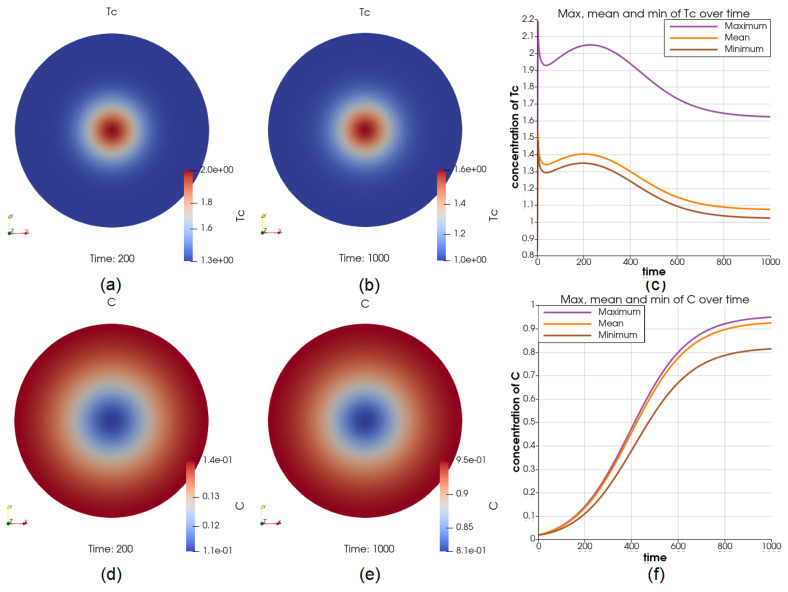
Illustrations for the case of a source of $T_{c}$ in the middle. Part (**a**,**b**) show the value of $T_{c}$ at t=200 and t=1000, respectively; (**c**) shows the dynamics of the maximum and minimum of $T_{c}$, depicting that the value of $T_{c}$ in the middle is constantly higher than it on the boundary by a considerable amount; the cancer cell concentration at t=200 and t=1000 are shown in (**d**,**e**); the value of the maximum and minimum of *C* over the whole time interval is shown in (**f**), where the growth of cancer cells in the middle are inhibited strongly by the higher concentration of $T_{c}$.

We also simulated the condition of a constant source of Cytotoxic and NK cells $T_{c}$ in the middle of the tumor. The source has resulted in a roughly 0.6 higher concentration of $T_{c}$ at all times, see Figure 8a,b for two time points t=200 and t=1000 and Figure 8c for the comparison of the maximum and minimum of $T_{c}$ in the whole time interval. At t=200, $V=4.87V_{0}$ (comparing with the reference case when $V=5.48V_{0}$), *C* has more than 20% higher concentration on the boundary and ranges between 0.11 and 0.14, see Figure 8d. At t=1000, $V=32.30V_{0}$ (comparing with the reference case when $V=34.6V_{0}$), *C* has a lower concentration in the middle and ranges between 0.81 and 0.95, see Figure 8e. The dynamics of *C* at the center and on the boundary are displayed in Figure 8f. The concentration of *C* is approaching its steady-state value of 1 much slower in the middle of the tumor.

### 3.6. The Case of a Positive Source of $T_{c}$ on the Boundary

**Figure 9:**
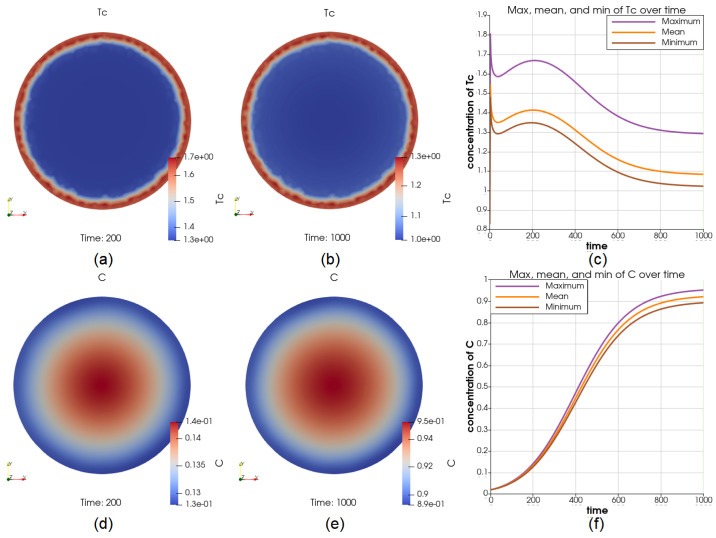
Illustrations for the case of a constant source of $T_{c}$ on the boundary. Su-figures (**a**,**b**) shows the value of $T_{c}$ at t=200 and t=1000, respectively; (**c**) shows the dynamics of the maximum and minimum of $T_{c}$, depicting that the value of $T_{c}$ on the boundary is constantly higher than it is elsewhere; the cancer cell concentration at t=200 and t=1000 are shown in (**d**,**e**); the value of the maximum and minimum of *C* over the whole time interval is shown in (**f**), where the growth of cancer cells, especially near the boundary, are inhibited strongly by the higher concentration of $T_{c}$.

We have simulated the condition of a constant source of cytotoxic and NK cells $T_{c}$ on the boundary of the tumor. The source has resulted in a roughly 33% higher concentration of this cell type over the boundary at all times; see Figure 9a,b for two time points t=200 and t=1000 and Figure 9c for the comparison of the maximum and minimum of $T_{c}$ in the whole time interval. At t=200, $V=4.77V_{0}$ (comparing with the reference case when $V=5.48V_{0}$), *C* has a lower concentration over the boundary and ranges between 0.127 and 0.142 (significantly lower than 0.16 in the reference case), see Figure 9d. At t=1000, $V=32.00V_{0}$ (comparing with the reference case when $V=34.6V_{0}$), *C* has a higher concentration in the middle and ranges between 0.89 and 0.95, see Figure 9e. The dynamics of *C* at the center and on the boundary are displayed in Figure 9f.

### 3.7. The Case of a Positive Source of M on the Boundary

**Figure 10:**
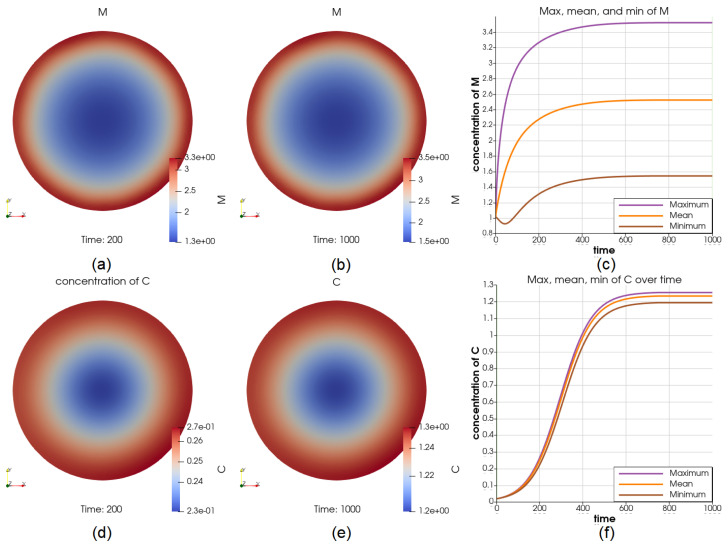
Illustrations for the case of a source of *M* on the boundary. Sub-figure (**a**,**b**) show the value of $T_{c}$ at t=200 and t=1000, respectively; (**c**) show the dynamics of the maximum and minimum of *M*, depicting that the value of *M* on the boundary is substantially higher than it is elsewhere; the cancer cell concentration at t=200 and t=1000 are shown in (**d**,**e**); the value of the maximum and minimum of *C* over the whole time interval is shown in (**f**). We can see that the cancer cell concentrations approach different steady-state values, positively related to the concentrations of Macrophages.

We have also simulated a constant source of macrophages *M* on the boundary of the tumor. The source has resulted in up to 133% higher concentration of this cell type over the boundary at most of the time points; see Figure 10a,b for two time points t=200 and t=1000 and Figure 10c for the comparison of the maximum and minimum of $T_{c}$ in the whole time interval. At t=200, $V=8.88V_{0}$ (comparing with the reference case when $V=5.48V_{0}$), *C* has a lower concentration over the boundary and ranges between 0.23 and 0.27 (significantly higher than 0.16 in the reference case), see Figure 10d. At t=1000, $V=42.09V_{0}$ (comparing with the reference case when $V=34.6V_{0}$), *C* has a higher concentration in the middle and ranges between 1.2 and 1.25, see Figure 10e. The dynamics of *C* at the center and on the boundary are displayed in Figure 10f. We can see that the cancer cell concentrations *C* approach different values, positively related to the concentrations of Macrophages *M*. Still, both *M* and *C* reach steady states.

**Remark** **1.** *Simulations associated with all three clusters of patients’ data from the ODE paper have been done. The results are qualitatively the same. Thus, only the results obtained from using data from cluster 1 have been shown in this paper.*

## 4. Discussion

Accumulating evidence demonstrates the critical roles of the tumor-infiltrating immune cells in tumors’ progression [28,29,30]. For example, it has been shown that cytotoxic T-cells are effector cells of adaptive immunity targeting osteosarcoma [31] and significantly affect the immune responses of osteosarcoma patients [32]. In addition, treatments with anti-tumor immunocompetence, such as NK cells and γδ T-cells appear to be effective for some osteosarcoma tumors [33,34].

Many mathematical models have been developed to study tumors’ initiation, and progression [35,36,37,38,39,40,41,42]. Some computational models include bone modeling, osteoblast cells, or osteosarcoma treatments [43,44,45,46,47]. Recently a data-driven ODE model for the progression of osteosarcoma tumors, which considers immune cells’ interactions with tumor cells, has been developed [12]. However, to the best of our knowledge, there is no PDE model for osteosarcoma tumors considering the immune cells and tumor cell interactions. We have extended a recent ODE model for the immune and cancer cell interactions in osteosarcoma tumors [12] by developing a bio-mechanical multiphase model. Using this PDE model, which includes spatial information, namely, cellular/molecular distributions, influxes, size, and shape of the domain, we have simulated different scenarios to investigate the effect of the location of immune cells and their influx on the location and concentration of cancer cells as well as the tumor’s growth.

We have used the solution of the mechanical part, which also considers the biological variables, to trigger the change in the domain. We assess the accuracy of the domain change from the number of cells that are a part of the solid tumor. The results shown in Table 3 suggest that the domain changes fall into a reasonable range. The model also captures the killing effect of cytotoxic T-cells well. Cancer cells become more concentrated in the middle of the tumor and depleted in the boundary when there is a constant source of cytotoxic cells on the boundary of the tumor (Figure 9), and vice versa if the $T_{c}$’s are more located in the middle of the tumor, Figure 8).

The model results emphasize the importance of the influx and the location of macrophages on the cancer cells’ concentration. As we simulated the influx of immune cells, we noticed a higher level of cancer cells with the influx of macrophages than any other immune cells. Importantly, suppose there is a constant source of macrophages on the boundary of the tumors. In that case, cancer cells are collocated with them on the boundary Figure 10d, implying that the concentration of macrophages is spatially positively related to the concentration of cancer cells. This relation cannot be observed through an ODE modeling of the tumor. These results agree with the observations of a high infiltration of macrophages in localized osteosarcoma tissues using immunohistochemical staining techniques [48].

The greatest challenge in this study was finding the parameter values of the Biot equations for osteosarcoma tumors. Ideally, those parameter values should be measured exclusively for osteosarcoma. However, if we find a value for general sarcomas, we will see it as a good approximation. As such examples, we found the bulk modulus *K*, shear modulus *G*, and hydraulic conductivity κ in [19]. We found the values of the rest of the parameters from [20]; these values were calculated using an artificial neural network for general tumors. Fortunately, using those parameters, the results we obtained are biologically sound. However, the results of the model should be experimentally and bio-medically validated.

## 5. Conclusions

To sum up, we have proposed a model that describes osteosarcoma tumor progression and explored the effect of immune cells infiltration in the cancer cells concentration. As we are providing this model as an open-source Python code, this model can be utilized by researchers and can be improved by considering more factors, such as angiogenesis and chemotactic and haptotactic effects, or be adjusted for other cancer types. It may also be used in developing a model to elaborate the mechanical tumor growth, which could be made possible with recently published MRI image sources and image processing results [49,50]. 

## Figures and Tables

**Table 1:** Variable names corresponding to $\left[X_{i}\right]$.

$\left[X_{i}\right]$	Variable Name	Biological Meaning (Concentration of)	Scaling Factor ${}^{†}$
$\left[X_{1}\right]$	$M_{n}$	Naive macrophages	$6.236\times 10^{6}$
$\left[X_{2}\right]$	*M*	Macrophages	$1.977\times 10^{7}$
$\left[X_{3}\right]$	$T_{n}$	Naive T cells	$4.926\times 10^{6}$
$\left[X_{4}\right]$	$T_{h}$	Helper T cells	$7.092\times 10^{6}$
$\left[X_{5}\right]$	$T_{r}$	Regulatory T cells	$3.675\times 10^{6}$
$\left[X_{6}\right]$	$T_{c}$	Cytotoxic T cells and NK cells	$2.292\times 10^{7}$
$\left[X_{7}\right]$	$D_{n}$	Naive dendritic cells	$4.826\times 10^{5}$
$\left[X_{8}\right]$	*D*	Dendritic cells	$9.865\times 10^{5}$
$\left[X_{9}\right]$	*C*	Cancer cells	$1.343\times 10^{10}$
$\left[X_{10}\right]$	*N*	Necrotic cells	$3.764\times 10^{8}$
$\left[X_{11}\right]$	$I_{\gamma }$	IFN-γ	0.868
$\left[X_{12}\right]$	$\mu_{1}$	TGF-β, IL-4, IL-10, and IL-13	21.510
$\left[X_{13}\right]$	$\mu_{2}$	IL-6 and IL-17	2.067
$\left[X_{14}\right]$	$H_{n}$	HMGB1	5.076

^†^ These are values associated with cluster 1 in the ODE paper [12].

**Table 2:** Mechanical parameter values.

Parameter	Name	Value
ϕ	Porosity	0.2
*K*	Bulk modulus	40,000 (Pa) [19] ${}^{†}$
*G*	Shear modulus	30,000 (Pa) [19] ${}^{†}$
α	Biot effective stress coefficient	0.7 [20] ${}^{‡}$
κ	Hydraulic conductivity	$6.9\times 10^{-14}$ ($m^{2}\cdot {\mathrm{Pa}}^{-1}\cdot s^{-1}$) [19] ${}^{†}$
*M*	Biot modulus	$2\times 10^{5}$ (Pa) [20] ${}^{‡}$
$D_{cell}$	Diffusion coefficient for cells	$3.6\times 10^{-8}\left({\mathrm{cm}}^{2}\cdot h^{-1}\right)$ [21]
$D_{cyto}$	Diffusion coefficient for cytokines	$5.2\times 10^{-5}\left({\mathrm{cm}}^{2}\cdot h^{-1}\right)$ [22]
$D_{H}$	Diffusion coefficient for HMGB1	$3.3\times 10^{-3}\left({\mathrm{cm}}^{2}\cdot h^{-1}\right)$ [23]

^†^ These values are for sarcoma, not necessarily osteosarcoma; ^‡^ These values are for generic tumors, evaluated through Artificial Neural Network (ANN) and then averaged among all reasonable values.

**Table 3:** The total number of cells *V*, the ratios $n=V/V_{0}$, the crude estimate of diameters $0.01\sqrt{n}$, the linear model’s prediction of diameters, and the diameter of the domain after we apply the solid displacement vector u, at different times.

t	Cell Number *V* ${}^{†}$	Ratio $n=V/V_{0}$	Crude Estimate $0.01\sqrt{n}$	Linear Model Estimate	Simulated Domain Diameter
0	33,726	1	0.01	0.01	0.01
10	24,264	0.785	0.0089	0.0087	0.0096
50	41,447	1.229	0.0111	0.0112	0.0102
100	71,778	2.128	0.0146	0.0153	0.0116
150	118,172	3.504	0.0187	0.0202	0.014
200	184,578	5.473	0.0234	0.0259	0.0176
250	273,510	8.110	0.0285	0.0324	0.0227
300	384,156	11.39	0.0337	0.0392	0.0291
350	510,781	15.14	0.0389	0.0459	0.0367
400	642,925	19.06	0.0437	0.0523	0.0451
450	768,233	22.78	0.0477	0.0578	0.0535
500	876,630	25.99	0.0510	0.0622	0.0614
550	963,109	28.56	0.0534	0.0656	0.0684
600	1,027,711	30.47	0.0552	0.0680	0.0743
650	1,073,622	31.83	0.0564	0.0697	0.0793
700	1,105,104	32.77	0.0572	0.0709	0.0834
750	1,126,165	33.39	0.0578	0.0716	0.0868
800	1,140,022	33.80	0.0581	0.0721	0.0897
850	1,149,042	34.07	0.0584	0.0724	0.0922
900	1,154,872	34.24	0.0585	0.0726	0.0944
950	1,158,622	34.35	0.0586	0.0728	0.0963
1000	1,160,215	34.40	0.0587	0.0728	0.098

^†^ These values are cell numbers in a 2D area. A scale of 10^6^ in a 2D area amounts to a scale of 10^9^ in a 3D volume.

**Figure A1:**
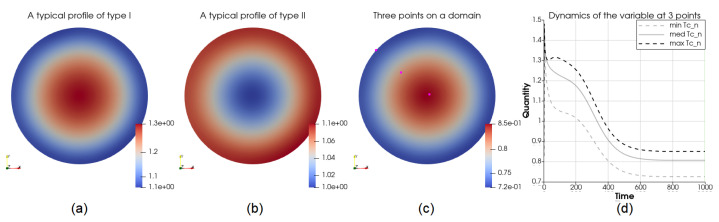
Common types of profiles in this paper. In part (**a**), the center has the highest density, the density reduces in the positive radial direction, and near the boundary takes its lowest value. This is because of the initial conditions of an influx through the boundary. Part (**b**) shows the opposite case. In part (**c**), three points are selected with different distance from the center. In part (**d**), the dynamics are plotted to show how much the density in the center is different from the boundary.

## Data Availability

Python scripts for computations and plotting the dynamical results are available here : https://github.com/ShahriyariLab/Bio-Mechanical-model-of-osteosarcoma-tumor-microenvironment-A-porous-media-approach, since November 2022.

## Appendix A. The ODE Model and Its Parameters

Here is the system of ODEs presented in [12] that models the biochemical interaction of key players of the osteosarcoma tumors given in Figure 1. In this paper, we have denoted the right hand-sides of this system by $f_{i}$’s. In the following equations, the proliferation and activation rates are denoted by λ’s, and the degradation and death rates are denoted by δ’s. In addition, the constant production rates of the cells are denoted by *A*’s. For the detail of the derivations of this system of ODEs, please see [12].

(A1) $$\begin{array}{cc} \frac{d\left[M_{N}\right]}{dt}= & A_{M_{N}}-\left(\lambda_{MI_{\gamma }}\left[I_{\gamma }\right]+\lambda_{M\mu_{1}}\left[\mu_{1}\right]\right)\left[M_{N}\right]-\delta_{M_{N}}\left[M_{N}\right], \end{array}$$

(A2) $$\begin{array}{cc} \frac{d\left[M\right]}{dt}= & \left(\lambda_{MI_{\gamma }}\left[I_{\gamma }\right]+\lambda_{M\mu_{1}}\left[\mu_{1}\right]\right)\left[M_{N}\right]-\delta_{M}\left[M\right], \end{array}$$

(A3) $$\begin{array}{ll} \frac{d\left[T_{N}\right]}{dt}= & A_{T_{N}}-\left(\lambda_{T_{h}M}\left[M\right]+\lambda_{T_{h}D}\left[D\right]\right)\left[T_{N}\right]\\ \; & -\lambda_{T_{r}\mu_{1}}\left[\mu_{1}\right]\left[T_{N}\right]\\ \; & -\left(\lambda_{T_{c}T_{h}}\left[T_{h}\right]+\lambda_{T_{c}M}\left[M\right]+\lambda_{T_{c}D}\left[D\right]\right)\left[T_{N}\right]-\delta_{T_{N}}\left[T_{N}\right], \end{array}$$

(A4) $$\begin{array}{cc} \frac{d\left[T_{h}\right]}{dt}= & \left(\lambda_{T_{h}M}\left[M\right]+\lambda_{T_{h}D}\left[D\right]\right)\left[T_{N}\right]-\left(\delta_{T_{h}T_{r}}\left[T_{r}\right]+\delta_{T_{h}\mu_{1}}\left[\mu_{1}\right]+\delta_{T_{h}}\right)\left[T_{h}\right], \end{array}$$

(A5) $$\begin{array}{cc} \frac{d\left[T_{r}\right]}{dt}= & \left(\lambda_{T_{r}\mu_{1}}\left[\mu_{1}\right]\right)\left[T_{N}\right]-\delta_{T_{r}}\left[T_{r}\right], \end{array}$$

(A6) $$\begin{array}{cc} \frac{d\left[T_{c}\right]}{dt}= & \left(\lambda_{T_{c}T_{h}}\left[T_{h}\right]+\lambda_{T_{c}M}\left[M\right]+\lambda_{T_{c}D}\left[D\right]\right)\left[T_{N}\right]\\ \; & -\left(\delta_{T_{c}T_{r}}\left[T_{r}\right]+\delta_{T_{c}\mu_{1}}\left[\mu_{1}\right]+\delta_{T_{c}}\right)\left[T_{c}\right], \end{array}$$

(A7) $$\begin{array}{cc} \frac{d\left[D_{N}\right]}{dt}= & A_{D_{N}}-\left(\lambda_{DC}\left[C\right]+\lambda_{DH}\left[H\right]\right)\left[D_{N}\right]-\delta_{D_{N}}\left[D_{N}\right], \end{array}$$

(A8) $$\begin{array}{cc} \frac{d\left[D\right]}{dt}= & \left(\lambda_{DC}\left[C\right]+\lambda_{DH}\left[H\right]\right)\left[D_{N}\right]-\left(\delta_{DC}\left[C\right]+\delta_{D}\right)\left[D\right], \end{array}$$

(A9) $$\begin{array}{cc} \frac{d\left[C\right]}{dt}= & \left(\lambda_{C}+\lambda_{C\mu_{1}}\left[\mu_{1}\right]+\lambda_{C\mu_{2}}\left[\mu_{2}\right]\right)\left[C\right]\left(1-\frac{\left[C\right]}{C_{0}}\right)\\ \; & -\left(\delta_{CT_{c}}\left[T_{c}\right]+\delta_{CI_{\gamma }}\left[I_{\gamma }\right]+\delta_{C}\right)\left[C\right], \end{array}$$

(A10) $$\begin{array}{cc} \frac{d\left[N\right]}{dt}= & \alpha_{NC}\left(\delta_{CT_{c}}\left[T_{c}\right]+\delta_{CI_{\gamma }}\left[I_{\gamma }\right]+\delta_{C}\right)\left[C\right]-\delta_{N}\left[N\right], \end{array}$$

(A11) $$\begin{array}{cc} \frac{d\left[I_{\gamma }\right]}{dt}= & \lambda_{I_{\gamma }T_{h}}\left[T_{h}\right]+\lambda_{I_{\gamma }T_{c}}\left[T_{c}\right]-\delta_{I_{\gamma }}\left[I_{\gamma }\right], \end{array}$$

(A12) $$\begin{array}{cc} \frac{d\left[\mu_{1}\right]}{dt}= & \lambda_{\mu_{1}T_{h}}\left[T_{h}\right]+\lambda_{\mu_{1}M}\left[M\right]+\lambda_{\mu_{1}C}\left[C\right]-\delta_{\mu_{1}}\left[\mu_{1}\right], \end{array}$$

(A13) $$\begin{array}{cc} \frac{d\left[\mu_{2}\right]}{dt}= & \lambda_{\mu_{2}T_{h}}\left[T_{h}\right]+\lambda_{\mu_{2}M}\left[M\right]+\lambda_{\mu_{2}C}\left[C\right]-\delta_{\mu_{2}}\left[\mu_{2}\right], \end{array}$$

(A14) $$\begin{array}{cc} \frac{d\left[H\right]}{dt}= & \lambda_{HM}\left[M\right]+\lambda_{HD}\left[D\right]+\lambda_{HN}\left[N\right]-\delta_{H}\left[H\right]. \end{array}$$

**Table A1:** Non-dimensional initial conditions for each cluster.

Cluster	$M_{N}/M_{N}^{\infty }$	$M/M^{\infty }$	$T_{N}/T_{N}^{\infty }$	$T_{h}/T_{h}^{\infty }$	$T_{r}/T_{r}^{\infty }$	$T_{c}/T_{c}^{\infty }$	$D_{N}/D_{N}^{\infty }$
1	2.367	1.005	0.019	0.794	0.764	0.828	1.122
2	0.954	0.753	1.299	1.451	2.313	0.062	0.071
3	0.866	1.104	0.572	0.340	0.484	0	1.643
	$D/D^{\infty }$	$C/C^{\infty }$	$N/N^{\infty }$	$I_{\gamma }/I_{\gamma }^{\infty }$	$\mu_{1}/\mu_{1}^{\infty }$	$\mu_{2}/\mu_{2}^{\infty }$	$H/H^{\infty }$
1	0	0.020	0.160	2.394	1.104	1.806	1.059
2	0.693	0.005	0.018	0.859	1.307	3.259	0.988
3	0	0.014	0.0008	0.276	1.030	1.296	1.284

**Table A2:** Non-dimensional parameter values for each cluster, the sources can be found in the ODE paper [12].

Parameter	Cluster 1	Cluster 2	Cluster 3
${\bar{\lambda }}_{MI_{\gamma }}$	4.3649 $\times 10^{-3}$	8.4234 $\times \;10^{-4}$	2.8083 $\times \;10^{-3}$
${\bar{\lambda }}_{M\mu_{1}}$	1.0635 $\times \;10^{-2}$	1.4158 $\times \;10^{-2}$	1.2192 $\times \;10^{-2}$
${\bar{\lambda }}_{T_{h}M}$	3.3434 $\times \;10^{-2}$	1.9270 $\times \;10^{-2}$	2.2194 $\times \;10^{-2}$
${\bar{\lambda }}_{T_{h}D}$	1.0963 $\times \;10^{1}$	7.3778	9.8325
${\bar{\lambda }}_{T_{r}\mu_{1}}$	6.3 $\times \;10^{-2}$	6.3 $\times \;10^{-2}$	6.3 $\times \;10^{-2}$
${\bar{\lambda }}_{T_{c}T_{h}}$	6.1171	2.3846	2.8415
${\bar{\lambda }}_{T_{c}M}$	1.7478	1.2263	1.4683
${\bar{\lambda }}_{T_{c}D}$	1.1463 $\times \;10^{1}$	9.3900	1.3011 $\times \;10^{1}$
${\bar{\lambda }}_{DC}$	4.0114 $\times \;10^{-1}$	4.8942 $\times \;10^{-1}$	5.9472 $\times \;10^{-1}$
${\bar{\lambda }}_{DH}$	4.1518 $\times \;10^{-1}$	4.2621 $\times \;10^{-1}$	4.1729 $\times \;10^{-1}$
$\lambda_{C}$	1.662 $\times \;10^{-2}$	1.662 $\times \;10^{-2}$	1.662 $\times \;10^{-2}$
${\bar{\lambda }}_{C\mu_{1}}$	3.7101 $\times \;10^{-4}$	3.5910 $\times \;10^{-4}$	4.0692 $\times \;10^{-4}$
${\bar{\lambda }}_{C\mu_{2}}$	7.1405 $\times \;10^{-4}$	6.3207 $\times \;10^{-4}$	5.7910 $\times \;10^{-4}$
${\bar{\lambda }}_{I_{\gamma }T_{h}}$	6.3095	1.1946 $\times \;10^{1}$	4.1848
${\bar{\lambda }}_{I_{\gamma }T_{c}}$	2.6961 $\times \;10^{1}$	2.1324 $\times \;10^{1}$	2.9085 $\times \;10^{1}$
${\bar{\lambda }}_{\mu_{1}T_{h}}$	1.8813 $\times \;10^{2}$	1.1491 $\times \;10^{2}$	1.0315 $\times \;10^{2}$
${\bar{\lambda }}_{\mu_{1}M}$	1.0751 $\times \;10^{2}$	1.1818 $\times \;10^{2}$	1.0661 $\times \;10^{2}$
${\bar{\lambda }}_{\mu_{1}C}$	1.9184 $\times \;10^{2}$	2.5440 $\times \;10^{2}$	2.7772 $\times \;10^{2}$
${\bar{\lambda }}_{\mu_{2}T_{h}}$	1.2313	6.8806 $\times \;10^{-1}$	6.0936 $\times \;10^{-1}$
${\bar{\lambda }}_{\mu_{2}M}$	1.4073	1.4153	1.2595
${\bar{\lambda }}_{\mu_{2}C}$	2.5113	3.0467	3.2811
${\bar{\lambda }}_{HM}$	4.4046	5.8355	1.8254
${\bar{\lambda }}_{HD}$	3.6107	5.5856	2.0218
${\bar{\lambda }}_{HN}$	5.0685 $\times \;10^{1}$	4.7279 $\times \;10^{1}$	5.4853 $\times \;10^{1}$
$\delta_{M_{N}}$	6.93 $\times \;10^{-1}$	6.93 $\times \;10^{-1}$	6.93 $\times \;10^{-1}$
$\delta_{M}$	1.5 $\times \;10^{-2}$	1.5 $\times \;10^{-2}$	1.5 $\times \;10^{-2}$
$\delta_{T_{N}}$	4.2 $\times \;10^{-4}$	4.2 $\times \;10^{-4}$	4.2 $\times \;10^{-4}$
${\bar{\delta }}_{T_{h}T_{r}}$	6.6404	3.1732	5.0991
${\bar{\delta }}_{T_{h}\mu_{1}}$	4.1253	3.9929	4.5246
$\delta_{T_{h}}$	2.31 $\times \;10^{-1}$	2.31 $\times \;10^{-1}$	2.31 $\times \;10^{-1}$
$\delta_{T_{r}}$	6.3 $\times \;10^{-2}$	6.3 $\times \;10^{-2}$	6.3 $\times \;10^{-2}$
${\bar{\delta }}_{T_{c}T_{r}}$	1.1671 $\times \;10^{1}$	5.5771	8.9620
${\bar{\delta }}_{T_{c}\mu_{1}}$	7.2505	7.0179	7.9524
$\delta_{T_{c}}$	4.06 $\times \;10^{-1}$	4.06 $\times \;10^{-1}$	4.06 $\times \;10^{-1}$
$\delta_{D_{N}}$	1.664	1.664	1.664
${\bar{\delta }}_{DC}$	5.3932 $\times \;10^{-1}$	6.3864 $\times \;10^{-1}$	7.3501 $\times \;10^{-1}$
$\delta_{D}$	2.77 $\times \;10^{-1}$	2.77 $\times \;10^{-1}$	2.77 $\times \;10^{-1}$
${\bar{\delta }}_{CT_{c}}$	1.2269 $\times \;10^{-2}$	9.6574 $\times \;10^{-3}$	8.4017 $\times \;10^{-3}$
${\bar{\delta }}_{CI_{\gamma }}$	4.5923 $\times \;10^{-3}$	6.4192 $\times \;10^{-3}$	8.4660 $\times \;10^{-3}$
$\delta_{C}$	3.0078 $\times \;10^{-4}$	1.0390 $\times \;10^{-3}$	2.4530 $\times \;10^{-4}$
$\delta_{N}$	4.5935 $\times \;10^{-1}$	4.8360 $\times \;10^{-1}$	1.1137 $\times \;10^{-1}$
$\delta_{I_{\gamma }}$	3.327 $\times \;10^{1}$	3.327 $\times \;10^{1}$	3.327 $\times \;10^{1}$
$\delta_{\mu_{1}}$	4.8748 $\times \;10^{2}$	4.8748 $\times \;10^{2}$	4.8748 $\times \;10^{2}$
$\delta_{\mu_{2}}$	5.15	5.15	5.15
$\delta_{H}$	5.87 $\times \;10^{1}$	5.87 $\times \;10^{1}$	5.87 $\times \;10^{1}$
${\bar{A}}_{M_{N}}$	7.4055 $\times \;10^{-1}$	7.0151 $\times \;10^{-1}$	7.1382 $\times \;10^{-1}$
${\bar{A}}_{T_{N}}$	1.0581 $\times \;10^{2}$	1.7917	3.1561
${\bar{A}}_{D_{N}}$	3.3325	2.3958	2.4867
$\alpha_{M_{N}M}$	3.1701	5.6721 $\times \;10^{-1}$	1.3878
$\alpha_{T_{N}T_{h}}$	1.4396	1.8848 $\times \;10^{-1}$	8.8053 $\times \;10^{-2}$
$\alpha_{T_{N}T_{r}}$	7.4588 $\times \;10^{-1}$	8.2864 $\times \;10^{-2}$	1.0267 $\times \;10^{-1}$
$\alpha_{T_{N}T_{c}}$	4.6531	3.0144 $\times \;10^{-2}$	1.3172 $\times \;10^{-1}$
$\alpha_{D_{N}D}$	2.0440	7.9922 $\times \;10^{-1}$	8.1299 $\times \;10^{-1}$

## Appendix B. Details about Discretization of the PDEs

We consider a partition $T_{h}$ of $\Omega^{t}$ into triangular elements and let ${\left\{P_{j}\right\}}_{j=1}^{N_{h}}$ be the set of all the interior nodes of the triangulation (where $N_{h}$ is the number of interior nodes). Let $P_{r}$ denote the standard space of polynomials of total degree less than or equal to *r*. We introduce the space of finite elements

(A15) $$X_{h}^{r}=\left\{g\in C^{0}\left(\bar{\Omega }\right):g{|}_{\tau }\in P_{r},\forall \tau \in T_{h}\right\},$$

that is the space of globally continuous functions that are polynomials of degree *r* on the single triangles of the triangulation $T_{h}$. We also define

(A16) $$Q_{h}=\left\{g\in X_{h}^{1}:g{|}_{\partial \Omega }=0\right\}.$$

The spaces $X_{h}^{r}$ and $Q_{h}$ are suitable for the approximation of $H^{1}\left(\Omega \right)$ and $H_{0}^{1}\left(\Omega \right)$, respectively [51]. The space $V_{h}$ is selected to be ${\left(X_{h}^{1}\right)}^{3}$. Denote the time step size by *k*, that is, k=T/N for some positive integer *N*, and $t_{n}=nk$ for n=0,1,…,N. Let $u^{n}=u\left(t_{n}\right)$ and $p^{n}=p\left(t_{n}\right)$ and let $U^{n}$ and $P^{n}$ be their approximations, respectively. The fully discrete approximation for the mechanical problem is: find $U^{n}\in V_{h}$ and $P^{n}\in Q_{h}$ for n=1,2,…,N and Lagrange multipliers $\lambda_{i}$ for i=1,2,3 such that

(A17) $$\begin{array}{cc} a\left(U^{n},v_{h}\right)+\left(\nabla P^{n},v_{h}\right)+\left(K\nabla V^{*n},v_{h}\right)-\sum_{i=1}^{3}\lambda_{i}\left(v_{h},z_{i}\right)-\sum_{i=1}^{3}\omega_{i}\left(u_{h},z_{i}\right)=0,\; & \end{array}$$

(A18) $$\begin{array}{cc} k^{-1}\left(c_{0}\left(P^{n}-P^{n-1}\right),q_{h}\right)+k^{-1}\left(\alpha \nabla \cdot \left(U^{n}-U^{n-1}\right),q_{h}\right)+\left(\kappa \nabla P^{n},\nabla q_{h}\right)=0,\; & \end{array}$$

for all basis function $v_{h}$ and $q_{h}$ of the spaces $V_{h}$ and $Q_{h}$. Then we solve the biological problem. Enriching the usual piece-wise linear function spaces using the bubble elements is a simple way of reducing the instability [52], for the diffusion-advection problems in the advection-dominated case [53], without increasing the size of the problem significantly [54,55].

We define

(A19) $$B^{\left(l\right)}=\mathrm{span}\left\{b_{\tau }^{l},\tau \in T_{h}\right\},$$

where $b_{\tau }^{l}$ are the *l*-th bubble functions. In this paper, we used the case r=1 and l=3, thus we define the bubble enriched piece-wise linear function spaces as

(A20) $$B=X_{h}^{1}⊕B^{\left(3\right)}.$$

Therefore, the finite element space of the biological problem is

(A21) $$X_{h}={\left(B\right)}^{14}.$$

The fully discrete approximation for the biological problem is: find $X^{n}\in {\left(H^{1}\left(\Omega \right)\right)}^{14}$ for n=1,2,…,N, such that

(A22) $$k^{-1}\left({\left[X\right]}^{n}-{\left[X\right]}^{n-1},\Xi_{h}\right)+\left(D\nabla {\left[X\right]}^{n},\nabla \Xi_{h}\right)+\left(b{\left[X\right]}^{n}\kappa \Delta p,\Xi_{h}\right)=(f,\Xi_{h}),$$

for all basis functions $\Xi_{h}$ of the space $X_{h}$. For the specific form of cubic bubble functions or a detailed introduction of bubble elements, the readers can see Chapter 9 of [56].

## Appendix C. Reports of Different Experiments

The following two types of profiles frequently show up when we plot the solution of the equations. If the variable has a higher value in the middle and a lower value over the boundary, because of different settings, as illustrated in Figure A1a, we call the profile of type I, or profile I. If, on the contrary, the variable has a higher value over the boundary and a lower value in the middle, as illustrated in Figure A1b, we call the profile of type II or profile II. Figure A1c,d show the three different locations on the domain we investigated and the dynamics of a variable at the three locations.

**Table A3:** A report of different experiments using no-flux boundary condition.

Initial Condition	Difference Level of IC (*max/min*)	*C* at t=200, (Relative Difference ${}^{†}$ in *C*)	*V*, (Relative Difference ${}^{†}$ in Relevant $X_{i}$), and Others
Ref. Case, (case Section 3.1) Uniform	1	C=0.16	$V=5.48V_{0}$
More $T_{h}$ around the boundary	13	(<1%)	$T_{h}$ profile evened out within t=2.5
More $T_{c}$ around the boundary	11	(<1%)	$T_{c}$ profile evened out within t=2.5
More $T_{c}$ in the middle	11	(<1%)	$T_{c}$ profile evened out within t=2.5
More *M* and $T_{c}$ in the middle, (case Section 3.2)	11, both	C∈[0.17,0.19], profile I (Figure A1a)	$V=6.25V_{0}$, *M* maintained profile I, $max\left(T_{c}\right)/min\left(T_{c}\right)=2$ at t=2.5, profile changed from I to II, then evened out
More *M* in the middle	11	Profile I	$V=6.26V_{0}$, *M* maintained profile I
More $M_{n}$ on the boundary	30	(<1%)	$V=5.68V_{0}$, $M_{n}$ profile evened out at about t=10

^†^ The relative difference is (*max* − *min*)/*min*.

**Table A4:** A report of different experiments using uniform initial condition.

Boundary Condition ($\alpha_{i}=1$, $\alpha_{i}\left[{\hat{X}}_{i}\right]=1$ by Default)	$\alpha_{i}$, $\alpha_{i}\left[{\hat{X}}_{i}\right]$	*C* at t=200, (Relative Difference in *C*)	*V*, (Relative Difference in Relevant $X_{i}$), and Others
Robin for all 5 cell types ${}^{†}$	-	-	(<0.1%), for all $X_{i}$
Robin for all	$\alpha_{i}\left[{\hat{X}}_{i}\right]=10$	maintained, (15%)	$V=5.53V_{0}$, *M* maintained profile I
Robin for $T_{h}$	$\alpha_{4}\left[{\hat{X}}_{4}\right]=10$	(0.1%)	-
Robin for $T_{c}$	$\alpha_{6}\left[{\hat{X}}_{6}\right]=1000$	C=0.16	$V=5.46V_{0}$, $T_{c}$ is 1% more on the boundary
Robin for *M*	-	-	(<1%)
Robin for *M*	$\alpha_{2}\left[{\hat{X}}_{2}\right]=0.5$	-	(<1%)
Robin for *M*	$\alpha_{2}\left[{\hat{X}}_{2}\right]=1000$	Profile II (Figure A1b), (>10%)	$V=7.89V_{0}$, *M* is 200% more on the boundary
Robin for $T_{c}$	-	-	(<0.1%)
Robin for all	$\alpha_{i}=10$	C=0.1543, (<1%)	$V=5.45V_{0}$, (≤0.1%)
Robin for all, (case Section 3.3)	$\alpha_{i}=100$, $\alpha_{i}\left[{\hat{X}}_{i}\right]=1$	C∈[0.142,0.147], Profile I	$V=5.09V_{0}$, ([5%,15%], for all *i*)
Robin for $T_{c}$	$\alpha_{6}=100$	-	(<1%)
Robin for $T_{h}$	$\alpha_{4}=100$	-	(<1%)
Robin for *M*	$\alpha_{2}=100$	0.151, (<1%)	$V=5.09V_{0}$, M∈[0.71,0.82], profile I

^†^ The five cell types are *M*, *T_h_*, *T_r_* , *T_c_*, and *D_n_*. Here *i* = 2, 4, 5, 6, 7.

**Table A5:** A report of different experiments using uniform initial condition and no-flux boundary condition.

Source	Location, Max Magnitude	*C* at t=200, (Relative Difference in *C*)	*V*, (Relative Difference in Relevant $X_{i}$), and Others
Gaussian for *M*, (case Section 3.4)	In the middle, 0.1	Profile I, (20%)	$V=6.70V_{0}$, (300%)
Gaussian for *C*	In the middle, 0.1	-	Nonlinear solver collapsed at t=7.5
Gaussian for *C*	In the middle, 0.01	-	Nonlinear solver collapsed at t=27.5
Gaussian for $T_{c}$, (case Section 3.5)	In the middle, 10	C∈[1.1,1.4], profile II	$V=4.87V_{0}$, (20%), profile I
Gaussian for $M_{n}$	In the middle, −4	-	Nonlinear solver collapsed at t=70
Step function for $T_{c}$	On the boundary, 1	Profile I, (<5%)	$V=5.29V_{0}$, $T_{c}\in \left[1.3,1.4\right]$, more on the boundary
Step function for $T_{c}$, (case Section 3.6)	On the boundary, 4	Profile I, (10%)	$V=4.75V_{0}$, $T_{c}\in \left[1.3,1.7\right]$, more on the boundary
Step function for $T_{h}$	On the boundary, 1	C=1.573, (<1%)	$V=5.57V_{0}$, $T_{h}$ is more on the boundary, (8%)
Step function for *M*	On the boundary, 1	C∈[0.88,1.1], profile II	$V=34.4V_{0}$, M∈[5.5,25], more on the boundary
Step function for *M*, (case Section 3.7)	On the boundary, 0.1	C∈[0.23,0.27], profile II	$V=8.88V_{0}$, M∈[1.3,3.3], more on the boundary
